# The introduction of biosimilars of low molecular weight heparins in Europe: a critical review and reappraisal endorsed by the Italian Society for Haemostasis and Thrombosis (SISET) and the Italian Society for Angiology and Vascular Medicine (SIAPAV)

**DOI:** 10.1186/s12959-017-0136-2

**Published:** 2017-05-10

**Authors:** Davide Imberti, Marco Marietta, Hernan Polo Friz, Claudio Cimminiello

**Affiliations:** 1 Haemostasis and Thrombosis Center, Internal Medicine Department, Piacenza Hospital, Via Taverna 49, Piacenza, Italy; 2 Department of Oncology and Hematology, Section of Hematology, University of Modena and Reggio Emilia, Modena, Italy; 3 Internal Medicine, Medical Department, Vimercate Hospital, via Santi Cosma e Damiano 10, 20871 Vimercate, Italy; 4 Studies and Research Center of the Italian Society of Angiology and Vascular Pathology (Società Italiana di Angiologia e Patologia Vascolare, SIAPAV), via Gorizia 22, 20144 Milan, Italy

**Keywords:** Low molecular weight heparin, Biosimilar pharmaceuticals, Evidence-based practice, Therapeutic equivalency, Venous Thromboembolism

## Abstract

Recently, the European Medicines Agency (EMA) authorized the introduction and marketing of Thorinane® and Inhixa®, biosimilars of the Low Molecular Weight Heparin (LMWH) enoxaparin. The authorization path is considerably different from the guidelines published by the EMA in 2009, as well as from the recommendations from the International Society on Thrombosis and Haemostasis published in 2013. Indeed, both of them recommended that LMWHs biosimilars therapeutic equivalence should be demonstrated in at least one adequately designed clinical trial. Shortly after enoxaparin biosimilars approval, EMA published a revised version of its guideline, no longer requiring the execution of a clinical study in patients at risk of venous thromboembolism.

Also the assessment of safety shows some relevant flaws, as it relies only on a 20 healthy volunteers study, clearly underpowered to draw any conclusions about the safety profile of the drug.

In our opinion, the approach taken by EMA for approval of enoxaparin biosimilars raises serious concerns about their actual, clinical “similarity”.

On these grounds, with the endorsement of the Italian Society for Haemostasis and Thrombosis (SISET) and the Italian Society for Angiology and Vascular Medicine (SIAPAV), we elaborated the present document aimed at reviewing and reappraising some critical points regarding the introduction of biosimilars of LMWH in Europe.

Moreover, we would strongly advise the Italian National Health Authorities not to entrust safety assessment to the post-marketing surveillance only, but to promote well designed and powered studies aimed at establish the actual efficacy and safety of LMWH biosimilars.

## Background

Low molecular weight heparins (LMWHs) are animal-derived products obtained by chemical or enzymatic depolymerization of unfractionated heparin. The efficacy and safety of LMWHs have been demonstrated in large randomized clinical trials and evidence based guidelines recommend their use for the prevention and treatment of venous and arterial thromboembolic events [1, 2].

Biological drugs are pharmaceutical products obtained by extraction from biological tissues or from biotechnological processes, constituted of larger molecules with a complex structure, thus differing from conventional small molecule medications [2]. The term “biosimilars” is used to qualify products developed to be similar to an original biological drug. Biosimilars are much more complicated to develop than a generic version of small molecule drugs and this is also true for LMWHs [3].

In the past years, patents of some LMWH have gradually expired and several copies of LMWHs have been produced and marketed in different countries. Recently, the European Commission granted a marketing authorization valid throughout the European Union for two biosimilars of LMWH enoxaparin (Thorinane and Inhixa).

The market accessibility of biosimilars is deemed to reduce costs to patients and social security systems. However, the introduction of biosimilar LMWHs have originated an intense debate since even minor differences between the biochemical and biological activities of biosimilar and originator LMWHs may have significant clinical consequences in terms of efficacy and safety [4].

Some serious concerns about the regulatory path adopted by EMA to authorize the introduction and marketing of Thorinane and Inhixa have led to the elaboration of the present document, endorsed by the Italian Society for Haemostasis and Thrombosis (SISET) and the Italian Society for Angiology and Vascular Medicine (SIAPAV), aimed at perform a review and a reappraisal of some critical points regarding the introduction of LMWH biosimilars in Europe.

## Main text

### Overview on guidelines, recommendations and requirements for the approval of biosimilars of LMWHs

Regulatory authorities from the United States of America (US) and Europe have taken different approaches to classify LMWH products. The Food and Drug Administration (FDA) considers LMWH semi-synthetic drugs while the European Medicines Agency (EMA) define them as biological products. Debates and controversies arising from these different positions determined the publication of several guidelines and position statements [2, 5–9]. Recommendations regarding the requirements that copies of LMWH must fulfill to be produced and marketed have been issued by the EMA [5]and the FDA [8, 9].

### FDA requirements

The FDA approved the first copy of enoxaparin in 2010. Innovator LMHWs were classified as drugs under the Abbreviated New Drug Application procedure proposed for requests for marketing authorization of small molecule chemical drugs, and requiring only the demonstration of bioequivalence through pharmacokinetic studies [8]. The FDA stated that the applicant for enoxaparin demonstrated the “sameness” compared to the branded LMWH enoxaparin, by meeting five criteria: (1) physical and chemical characteristics of enoxaparin; (2) nature of the source material and the method used to cleave the polysaccharide chains into smaller fragments; (3) nature and arrangement of components that constitute enoxaparin; (4) certain laboratory measurements of anticoagulant activity and (5) certain aspects of the drug’s effect in humans [8, 9].

### EMA guidelines

The EMA developed several guidelines and revisions on different aspects of the process of biosimilars LMWHs products approval.

In 2006, the EMA published the “Guideline on similar biological medicinal products containing biotechnology-derived proteins as active substance: quality issues (EMEA/CHMP/BWP/49348/2005)” [10]. Later, this document was replace by the “Guideline on similar biological medicinal products containing biotechnology-derived proteins as active substance: quality issues (revision 1). EMA/CHMP/BWP/247713/2012” [11]. Even though not specifically issued to regulate LMWHs biosimilars production, this guideline was used as reference to establish special quality aspects of biochemical LMWH characterization requirements.

In 2009, the EMA published the “Guidelines on non-clinical and clinical development of similar biological medicinal products containing low molecular weight heparin”EMEA/CHMP/BMWP/118264/2007 [5]. The document, currently in force, states as principle the need of a demonstration of the similar nature of the originator and the biosimilar in terms of safety and efficacy. Indeed, some specific criteria have to be fulfilled for a generic LMWH to be licensed, like: biochemical characterization; data on in vitro comparative bioassays (based on state of the art knowledge about clinically relevant pharmacodynamic effects of LMWH and including, at least, evaluations of anti-FXa and anti-FIIa activity); data on in vivo pharmacodynamic models comparing animal pharmacodynamic activity of the similar and the reference LMWH; data from at least one repeated dose toxicity study in a relevant species; phase I studies comparing the absorption and elimination characteristics and other pharmacodynamics tests such as Tissue Factor Pathway Inhibitor (TFPI) activity, as well as the ratio of anti-FXa and anti-FIIa activity. Moreover, the pharmacodynamic properties of biosimilars and branded LMWH must be compared in a randomized, single dose two way crossover study in healthy volunteers using subcutaneous administration, and, in case the originator product were also licensed for the intravenous or intra-arterial route, an additional comparative study should be performed via the intravenous route. In addition, the EMA guideline states that, since there is no clear correlation between pharmacodynamics parameters (anti-FXa or anti-FIIa) and clinical, a biosimilar LMWH should show equivalent efficacy and safety to a reference product approved in the EU. This therapeutic equivalence should be demonstrated in at least one adequately powered, randomized, double-blind, parallel group clinical trial. To demonstrate efficacy in the prevention of venous thromboembolism (VTE) in patients undergoing surgery with high VTE risk, the trial should be preferably conducted in major orthopedic surgery such as hip surgery and patients with hip fracture should be well represented. The study should be powered to show therapeutic equivalence on one of the two recommended endpoints and a central independent and blinded committee of experts should perform adjudication of VTE events.

Finally, the 2009 EMA guideline states that prelicensing safety data should be obtained in a number of patients sufficient to determine the adverse effect profiles of the test medicinal product, with comparative safety data from the efficacy trial being considered sufficient to provide an adequate pre-marketing safety database, and that a risk management programme plan in accordance with EU legislation and pharmacovigilance guidelines, with a particular focus on rare serious adverse events known to be associated with LMWHs such as Heparin-induced Thrombocytopenia Type II (HIT II, HITT) as well as anaphylactoid and anaphylactic reactions [5].

The EMA published a concept paper on the revision of the 2009 guideline in 2011: EMA/CHMP/BMWP/522386/2011 [12]. In the document, it was acknowledged that “the current guidence requires a comparative clinical trial demonstrating similar efficacy and safety” between the biosimilar and the reference LMWH in the prevention of VTE in patients undergoing major orthopaedic surgery, and was recommended a discussion whether a reduction in clinical data requirements could, in exceptional cases, be possible.

In 2014, the Committee for Medicinal Products for Human Use (CHMP) of the EMA issued the “Guideline on similar biological medicinal products” CHMP/437/04 Rev. 1 [13], with the purpose of describing the concept of similar biological medicinal products and to outline the general principles to be applied. CHMP experts concluded that the biosimilar approach is more difficult to apply to biological substances arising from extraction from biological sources and/or those for which little clinical and regulatory experience has been gained, like LMWHs, when compared to products that are highly purified and can be thoroughly characterized. Furthermore, this guideline states that a biosimilar should be highly similar to the reference medicinal product in physicochemical and biological terms and that any observed differences have to be duly justified with regard to their potential impact on safety and efficacy.

### International Society on thrombosis and Haemostasis (ISTH) recommendations

In 2013, the Scientific Subcommittee (SSC) on Control of Anticoagulation of the Scientific and Standardization Committee of the ISTH published an update summarizing the recommendations for the development of a biosimilar version of a branded LMWH [2]. The SSC of the ISTH recommends that the lack of significant differences between the biosimilar and originator LMWH should be demonstrated using an adequate study design, and that all results obtained in vitro, ex vivo and in clinical settings should adequately demonstrate the similarity or non-inferiority of the biosimilar LMWH relative to the originator LMWH and the confidence intervals should be defined using adequate statistical methods. Furthermore, the SSC of the ISTH clearly states that the efficacy and safety of a biosimilar LMWH should be demonstrated in comparison to the originator LMWH in clinical trials for every indication for which regulatory approval is sought. If biosimilar LMWHs claim to be as effective and safe as the originator products, a head to head comparison of the two LMWH preparations should be performed also in prospective, randomized, double blind clinical trials performed to show the non-inferiority of a biosimilar LMWH compared to the originator LMWH, in the most relevant clinical settings where LMWHs are indicated, like prophylaxis of postoperative venous thromboembolism, prophylaxis of venous thromboembolism in hospitalized patients with acute medical illness, treatment of acute deep vein thrombosis and pulmonary embolism, prevention of acute coronary events in patients with unstable or stable angina, prevention of acute coronary syndrome during and after percutaneous coronary intervention, extracorporeal circulation, and chronic haemodialysis [2].

### The authorization path for the approval of biosimilars of LMWHs in Europe

In July 2016 the EMA’s CHMP expressed a positive opinion for granting a marketing authorization to two biosimilars of enoxaparin sodium: Thorinane and Inhixa [14, 15]. Both European Public Assessment Reports (EPAR)s were first published on 26 October 2016. Thorinane’s application was received by the EMA on 6 February 2015 and the procedure started on 25 March 2015. Inhixa’s application was received on 27 May 2015 and the procedure started on 25 June 2015.

Both reports declared that the development programme of Thorinane and Inhixa had specifically considered the EU guidelines for similar biological medicinal products including the following specific guidelines for LMWH:

- CHMP Guideline on similar biological medicinal products containing biotechnology-derived proteins as active substance: quality issues (revision 1)(EMA/CHMP/BWP/247713/2012) [11]
- CHMP Guideline on Similar Biological Medicinal Products containing Biotechnology-Derived Proteins as Active Substance: Non-Clinical and Clinical Issues(EMEA/CHMP/42832/05) [16]
- Guideline on Non-Clinical and Clinical Development of Similar Biological Medicinal Products containing Low-Molecular-Weight-Heparins(EMEA/CHMP/BMWP/118264/2007) [5]
- Concept paper on the revision of the guideline on nonclinical and clinical development of similar biological medicinal products containing low molecular-weight heparins (EMA/CHMP/BMWP/522386/2011) [12]

Main conclusions reported by CHMP in the EPARs of Thorinane and Inhixa concerned non clinical aspects, clinical pharmacology, clinical efficacy and clinical safety. In general, they are the same for both products (Thorinane and Inhixa), since the rationale, authorization path and conclusions presented in both EPARs are practically identical [14, 15].

### The recent approval of biosimilars of LMWHs in Europe and November 2016 revision of the EMA guidelines

Relevant concerns arise from the analysis of the authorization path that led to the recent approval of biosimilars of LMWHs by EMA, and some critical points should be clarify.

With regards to the non-clinical aspects, although some differences in the content of link region (LR) were found between Thorinane and the reference product, Thorinane EPAR authors stated that “the Applicant provided justification that the LR region is a structural feature of Enoxaparin which has no known pharmacological role that directly or indirectly affects either Heparin or Enoxaparin molecules”. The same for Inhixa. No comparative or stand-alone toxicity studies were performed to compare Thorinane and Inhixa and the reference RMP, since “toxicology studies could be not required if the quality comparability investigations of Thorinane and the RMP (addressing physicochemical parameters/analytical characterization as well as biological/biochemical parameters and similarity in biological activity) yield the expected results and did not leave open unanswered questions.” The CHMP concluded that relevant assays were conducted and were not able to identify different immunogenic potential for Thorinane [14] and Inhixa [15] when compared to the reference medicinal product (RMP). Even though it was acknowledged that in vitro data with respect to immunogenicity have limitations, the most prominent safety concern associated with LMWHs, HP4 (Heparin Platelet Factor 4) complex binding was “most likely similar between the test and the RMP”, and from this “it was inferred that the risk for immunogenicity is most likely also similar”. This kind of inference must be better clarify, especially considering the 2008 so-called heparin crisis, where severe immune reactions were documented to be associated to the presence of oversulfated chondroitin sulfate (OSCS) as a result of a potential contamination during the extraction process of heparin from the animal source [17].

In the discussion on clinical efficacy, both Thorinane and Inhixa EPARs [14, 15] mentioned that “The EMA Guideline on non-clinical and clinical development of similar biological medicinal products containing low-molecular-weight-heparins (EMEA/CHMP/BMWP/118264/2007 + Draft Rev. 1) foresees a clinical study comparing efficacy and safety of the biosimilar candidate and the reference product unless evidence for similar efficacy and safety of the biosimilar and the reference product could be convincingly deduced from the comparison of their physicochemical characteristics, biological activity/potency, using sensitive, orthogonal and state-of-the-art analytical methods, and from comparison of their PD profiles.” Instead, the 2009 “Guideline on non-clinical and clinical development of similar biological medicinal products containing low-molecular-weight-heparins”, in force currently and at the time when Thorinane and Inhixa EPARs were released, clearly states that “since a clear correlation between surrogate PD parameters (anti FXa or anti FIIa) and clinical outcome has not been established”… “this therapeutic equivalence should be demonstrated in at least one adequately powered, randomized, double-blind, parallel group clinical trial”, describing in detail the characteristics of that trial, like design and clinical setting. Thus, though there was no clinical efficacy studies performed to support the biosimilarity claims by Thorinane nor Inhixa, EPARs authors concluded that “it was agreed that potential efficacy study would not be sensitive enough to reveal small differences between two similar enoxaparin- containing-products showing a similar PD profile”, and that “a stringent comparative quality documentation supported by a reduced (non-)clinical program was considered appropriate for showing equivalence of efficacy of LMWH”.

When discussing on clinical safety issues, in both Thorinane and Inhixa EPARs, CHMP acknowledged that “the presented clinical safety data derived from a comparative PK/PD study were too scarce to conclude on a comparable safety profile of test and reference medicinal products”, that “immunogenicity has not been comparatively assessed and initially” and that “the applicant did not present a strategy of *in vitro* and/or *in vivo* assays to allow for waiving of clinical safety data” [14, 15]. However, and surprisingly, the CHMP finally concluded that “the enhanced assay strategy provided by the applicant during the procedure gave reassurance that the most prominent safety concern associated with LMWHs, HP4 complex binding is most likely similar between both tested products, thus “In light of established biosimilarity on quality level, the remaining uncertainty that the safety profile of Thorinane and Clexane differs significantly was considered low enough to conclude on similarity” [17]. The same concept is expressed in the EPAR for Inhixa [11], and implies that surveillance and pharmocovigilance are the only tools to recognize potential safety issues, even though, as the case of surveillance of a biosimilar of enoxaparin in the US shows, they seems to present critical limitations [18].

A revision of the 2009 “Guidelines on non-clinical and clinical development of similar biological medicinal products containing low molecular weight heparin” was issued in November 2016 [19]. Concerning clinical efficacy, this revised guideline, expected to be coming into effect in June 2017, concludes that the evidence for similar efficacy should be derived from the similarity demonstrated in physicochemical, functional and pharmacodynamic comparisons, and that a dedicated comparative efficacy trial will be no longer considered necessary. With regards to clinical safety, the guideline states that whether “the impurity profile and the nature of excipients of the biosimilar do not create uncertainties with regard to their impact on safety/ immunogenicity, a safety/immunogenicity study may not be needed”.

Thus, this guideline represent a conceptual and operative radical change respect to the previous EMA’s guidelines, and does not seem their logical evolution. Moreover, we think that the timing of such a change deserves some attention.

The EMA issued the “Concept paper on the revision of the guideline on nonclinical and clinical development of similar biological medicinal products containing low-molecular-weight heparins” (EMA/CHMP/BMWP/522386/2011) in 2011 [12], recommending a discussion about including the possibility of a modification in clinical data requirements. A draft revision was issued in 2012, but the Revision 1 of the guideline that makes effective these major modifications was adopted by CHMP on November 10th 2016, and, as mentioned, will be coming into effect on June 1st 2017.

That is, in July 2016, when EPARs authorizing the marketing of Thorinane and Inhixa were completed, the 2009 EMEA/CHMP/BMWP/118264/2007 guideline was in force, and such a guideline stated that therapeutic equivalence should be demonstrated in at least one adequately designed clinical trial.

Therefore, both enoxaparin biosimilars have been approved by using criteria quite different (and less compelling) from those required from the EMA guideline in force at that time. In our opinion, the “fast-track” approach taken by EMA for approval of enoxaparin biosimilars raises serious concerns about their actual, clinical “similarity”, and can become a dangerous precedent for other drugs.

## Conclusions

The authorizative path adopted by EMA for the introduction of biosimilar LMWHs in Europe raises in our opinion some relevant concerns regarding efficacy and safety of these drugs.

As far as Thorinane® and Inhixa® is concerned, the approval by the EMA was based only on in vitro preclinical assays (acknowledging that in vitro data with respect to immunogenicity have limitations) and on the outcome of a clinical PK/PD study in 20 healthy volunteers.

This approach is in keeping with the conceptual approach of FDA, which considers copies of LMWHs mostly generic drugs rather than biosimilars. However, we are unable to find any strong evidence supporting the EMA’s recent position, so divergent from that advocated in the past by the same regulatory agency as well as from the recommendation of the ISTH. We think that the EMA should provide to the scientific community a more in-depth explanation of such a decision and of the rationale which led to concluding on biosimilarity for Thorinane® and Inhixa® that “in vitro preclinical assays as well as the outcome of the primary endpoints of the clinical PD study provided comprehensive information for characterisation of the biosimilar candidate to conclude similarity regarding efficacy” [14, 15].

Even stronger concerns are raised by the conclusions about safety, which are based just on a small-sized PK/PD study in healthy volunteers. Relevant to this, EMA itself acknowledges that data provided by this study are too scarce to conclude on a comparable safety profile, and entrusts the safety assessment to the post-marketing pharmacovigilance. The already cited study by Grammp et al. [18]provide interesting data to better understand how hazardous such an approach may result. These Authors compared the capabilities of claims databases and spontaneous reporting systems for monitoring the incidence of potential enoxaparin-related adverse effect (AE)s, including thrombocytopenia-related AEs, at the product-specific level, and to compare the attribution of all enoxaparin-related AEs in the FDA AE Reporting System (FAERS) database. The study found that claims data were useful for active surveillance of enoxaparin biosimilar products dispensed under pharmacy benefits but not for products administered under medical benefits. With enoxaparin, 10–35% of spontaneous reports were not attributable to a given manufacturer, and a ninefold increase in relative risk of an AE for a specific enoxaparin biosimilar could be overlooked because of the apparent underreporting to specific biosimilar manufacturers. Authors concluded that the current spontaneous reporting system will not distinguish product-specific safety signals for products distributed by multiple manufacturers, including biosimilars, and the upcoming introduction of biosimilars into the marketplace has highlighted current limitations within the data infrastructure [18]. Therefore, surveillance does not seems the final answer to LMWHs biosimilars safety concerns.

An interesting example on the scientific and regulatory debate related to biosimilars approval is represented by single-switch crossover or transition trials of biosimilar anti-TNF agents. Since no conclusive clinical trial data demonstrating the efficacy and safety of switching the therapy (originator to biosimilar) of stable patients are available, the Norwegian government decided to support a randomized, double-blind, parallel-group study, the NOR-SWITCH biosimilar study, to compare the originator infliximab with a biosimilar in patients with six immune-mediated inflammatory diseases [20].

Scientific societies such as ISTH and IUA [2, 7] have formulated recommendations about the criteria that LMWH biosimilars must fulfill to be authorized. These criteria were similar to, and even more strict than, those adopted by EMA until July to November 2016.

We think that the EMA’s change of course is not supported by strong evidences, and therefore we stay on the requirements already issued by the scientific societies ISTH and IUA, thus asking for more reliable clinical data about the efficacy and safety of biosimilar LMWHs before their marketing.

Efficacy and safety assessment of biosimilars of LMWH should not be only based on post-marketing surveillance. Instead, therapeutic equivalence should be demonstrated in at least one adequately powered, randomized, double-blind, parallel group clinical trial, preferably in the prevention of VTE in patients with high VTE risk, with adjudication of VTE events by a central independent and blinded committee of experts. Prelicensing safety data should be obtained in a number of patients sufficient to determine the adverse effect profiles of the test medicinal product, with comparative safety data from the efficacy trial being considered sufficient to provide an adequate pre-marketing safety database, mainly aimed to assess endpoints such immunogenic adverse effects.

In conclusion, we agree that the development of biosimilar drugs can be an effective strategy to contain pharmaceutical expenses, thus providing more people with a wider access to treatments that are becoming more and more expensive. However, this appreciable goal should pursued by means of strict procedures, shared between stakeholders and scientific community, always placing the patient’s safety in the first place.

We think that the approach taken by EMA for approval of enoxaparin biosimilars doesn’t fulfill these requirements, raises serious concerns about their actual, clinical “similarity”..

On these grounds, we would strongly advise the Italian National Health Authorities not to entrust safety assessment to the post-marketing surveillance only, but to promote well designed and powered studies aimed at establish the actual efficacy and safety of LMWH biosimilars, as already performed for other molecules [20].

## Abbreviations

AE's: Adverse effect
CHMP: Committee for Medicinal Products for Human Use
EMA: European Medicines Agency
EPAR's: European Public Assessment Reports
FDA: Food and Drug Administration
ISTH: International Society on Thrombosis and Haemostasis
LMWH: Low Molecular Weight Heparin
SSC: Scientific Subcommittee
VTE: Venous thromboembolism
